# The effect of social support on sports performance in high-level collegiate volleyball athletes: the chain mediating effect of emotional intelligence and athletic engagement

**DOI:** 10.3389/fpsyg.2026.1833306

**Published:** 2026-05-26

**Authors:** Tingxiao Zhang, Peiyuan Zhao, Yaxing Geng, Qize Shi, Tianrui Yang

**Affiliations:** School of Physical Education, Liaoning Normal University, Dalian, China

**Keywords:** athletic performance, emotional intelligence, exercise engagement, high-level volleyball athletes from universities, social support

## Abstract

**Aim:**

This study targeted elite collegiate volleyball athletes to examine the current status of their perceived social support, emotional intelligence, and sport engagement, and to explore the relationship between social support and athletic performance. Specifically, it tested the chain mediating effects of emotional intelligence and sport engagement in the relationship between social support and athletic performance.

**Methods:**

The Social Support Scale for Elite Collegiate Volleyball Athletes, the Emotional Intelligence Scale for Elite Collegiate Volleyball Athletes, and the Sport Engagement Scale for Elite Collegiate Volleyball Athletes were administered. Using a combination of online and offline surveys, questionnaires were distributed to athletes from 39 teams participating in the 2024–2025 Chinese University Volleyball League (Elite Division).

**Results:**

(1) Social support was significantly positively correlated with athletic performance (r = 0.767, *p* < 0.01); emotional intelligence was significantly positively correlated with athletic performance (r = 0.783, *p* < 0.01); and sport engagement was significantly positively correlated with athletic performance (r = 0.765, *p* < 0.01). All sub-dimensions of the variables were significantly positively correlated with athletic performance (*p* < 0.01), among which emotional intelligence showed the strongest correlation.(2) Social support was significantly positively correlated with emotional intelligence (r = 0.872, *p* < 0.01); social support was significantly positively correlated with sport engagement (r = 0.841, *p* < 0.01); and emotional intelligence was significantly positively correlated with sport engagement (r = 0.867, *p* < 0.01).(3) In the overall sample, emotional intelligence played a significant mediating role between social support and athletic performance (effect = 0.414, accounting for 54.415% of the total effect), and sport engagement also played a significant mediating role (effect = 0.344, accounting for 44.796% of the total effect).(4) Emotional intelligence and sport engagement jointly formed a significant chain mediating pathway between social support and athletic performance, namely: social support→emotional intelligence→sport engagement→athletic performance (effect = 0.126, accounting for 16.43% of the total effect, *p* < 0.01). This chain mediation effect was stronger and more stable among female athletes (female effect = 0.133, 15.983% of total effect) than among male athletes (male effect = 0.120, 14.652% of total effect).

**Conclusion:**

High-level college volleyball athletes receive certain social support and perform well in emotional intelligence and athletic engagement. There are age differences in social support, emotional intelligence, and athletic engagement. Social support, emotional intelligence, athletic engagement and their sub-dimensions are positively correlated with sports performance, among which emotional intelligence shows the strongest correlation. In addition, the three variables are positively correlated with each other. Emotional intelligence and athletic engagement each play a significant mediating role between social support and sports performance, and a chain mediating effect exists. The chain mediating effect is stronger and more stable in females than in males.

## 1 Introduction

Social support, defined as the emotional, informational, and tangible assistance individuals obtain from their social networks, is widely recognized as a critical external resource influencing psychological health and behavioral performance. For collegiate athletes facing the dual pressures of intensive training and academic demands, positive social support can reduce anxiety, enhance psychological resilience, and indirectly improve athletic performance (Ren, 2023; Chang, 2024). As an important external environmental resource, social support lays the foundation for the development of individual psychological resources and behavioral inputs. Meanwhile, social support exerts a more pronounced effect when individuals face adversity, offering them material and emotional support (House et al., 1988).

High-level athletic performance depends not only on physical ability and technical skills but also on psychological factors. As noted in Sport Management Research, psychological training helps athletes regulate mental states, enhance specific psychological competencies, reduce external interference, and unlock athletic potential. Exploring the psychological mechanisms of athletic performance thus carries important theoretical and practical significance (Zhou, 2009).

Emotional intelligence refers to the ability to perceive, understand, and regulate emotions, and acts as a key internal psychological resource for athletes. Previous studies have shown that emotional intelligence is positively associated with psychological resilience and can reduce competition anxiety, thereby improving performance (Yang, 2024). Sport engagement reflects athletes’ positive psychological states (vigor, dedication, absorption) and is a proximal predictor of athletic performance, with profound implications for athletes’ wellbeing and long-term development (Zhang, 2024; Wang Z., 2024). Sports commitment can also influence athletes’physical fitness and psychological status, and even exert direct effects on their future life trajectories and personal development (Long, 2024).

Prior research has confirmed stable relationships among these variables. Social support positively predicts emotional intelligence and sport engagement (Lu, 2024; Hu, 2025). In turn, emotional intelligence promotes higher levels of sport engagement by enhancing sustained effort and concentration (Wang Z., 2024).

However, existing studies have mostly examined these variables separately, lacking an integrated framework to clarify their chain mediation mechanism. Most focus on single pathways rather than the continuous transmission from external resources to internal psychological states and behavioral inputs.

To address this gap, the present study examines the relationships among social support, emotional intelligence, sport engagement, and athletic performance among elite collegiate volleyball athletes. We specifically test the chain mediating roles of emotional intelligence and sport engagement in the effect of social support on athletic performance. This study clarifies the internal psychological pathway, enriches sport psychology theory, and provides practical implications for psychological training and performance promotion among high-level athletes.

## 2 The chain mediating hypothesis model of social support and sports performance of high-level volleyball athletes in colleges and universities

### 2.1 The predictive effect of social support on sports performance of high-level volleyball athletes in colleges and universities

As the sum of emotional, informational, and substantive help that individuals obtain from their social relationship networks, social support has been widely proven to have a significant positive predictive effect on athletes’ sports performance. Support from multiple dimensions such as coaches, teammates, families, and organizations can not only provide athletes with material security and training resources but also alleviate their competitive anxiety and enhance their psychological resilience at the psychological level, thereby indirectly promoting the exertion of their competitive level (Ren, 2023; Chang, 2024). Existing studies have shown that social support can directly and positively predict the sports performance of college athletes in projects such as football, basketball, and taekwondo, and exhibit a stable promoting effect in different sports projects (Zhang, 2022; Hu, 2017; Qiu, 2015). Based on this, we propose Hypothesis H1: Social support has a positive and significant predictive effect on the sports performance of high-level college volleyball athletes.

### 2.2 The predictive effect of emotional intelligence on sports performance

Emotional intelligence, as an individual’s ability to recognize, understand, manage, and utilize emotional information, is regarded as an important psychological variable connecting the external environment and individual behavioral performance. In the context of competitive sports, athletes with high emotional intelligence can more effectively perceive their own and their teammates’ emotional states, adjust tactical cooperation in a timely manner, and maintain a stable psychological state under high-pressure situations, thereby improving their competitive performance (Mayer and Salovey, 1997; Lin, 2024). Existing studies have confirmed that emotional intelligence is significantly correlated with the psychological resilience, competitive state anxiety, and sports performance of college athletes, and has a direct positive predictive effect on sports performance (Yang, 2024; Zhang, 2003). Based on this, we propose Hypothesis H2: Emotional intelligence has a positive and significant predictive effect on the sports performance of high-level college volleyball athletes.

### 2.3 The predictive effect of athletic engagement on sports performance

Athletic engagement refers to the positive psychological states such as vitality, dedication, concentration, and enthusiasm displayed by athletes in training and competitions, and is a core proximal variable predicting sports performance. Athletes with high athletic engagement show stronger motivation, higher effort, and more persistent perseverance in training, and can more effectively transform training results into competitive performance (Lonsdale et al., 2007; Zhai et al., 2019). Existing studies have shown that athletic engagement can not only directly and positively predict the sports performance of athletes in projects such as Sanda and sports dance, but also play a complete mediating role between the coach-athlete relationship and sports performance (Liu, 2023; Chang, 2024). Based on this, we propose Hypothesis H3: Athletic engagement has a positive and significant predictive effect on the sports performance of high-level college volleyball athletes.

### 2.4 The correlation among social support, emotional intelligence, and athletic engagement

There is a close internal connection among social support, emotional intelligence, and athletic engagement. On the one hand, social support can significantly and positively predict athletes’ emotional intelligence level. A good social support environment provides athletes with rich opportunities for emotional communication and learning, helping them improve their abilities in emotional perception, expression, and regulation (Hu, 2025). On the other hand, social support can also significantly and positively predict athletes’ athletic engagement level. When athletes perceive support from coaches, teammates, and families, it will stimulate their intrinsic motivation and enhance their level of engagement in training and competitions (Lu, 2024; Bianco and Eklund, 2001). In addition, emotional intelligence also has a significant positive predictive effect on athletic engagement. Athletes with high emotional intelligence can better regulate negative emotions in training and competitions, maintain a positive attitude, and thus engage in sports more attentively and persistently (Wang Z., 2024). Based on this, we propose the following hypotheses: H4a: There is a significant positive correlation between social support and emotional intelligence; H4b: There is a significant positive correlation between social support and athletic engagement; H4c: There is a significant positive correlation between emotional intelligence and athletic engagement.

### 2.5 The prediction of the chain mediating effect of perceived family support, self-efficacy, and physical exercise behavior

**Figure 1:**
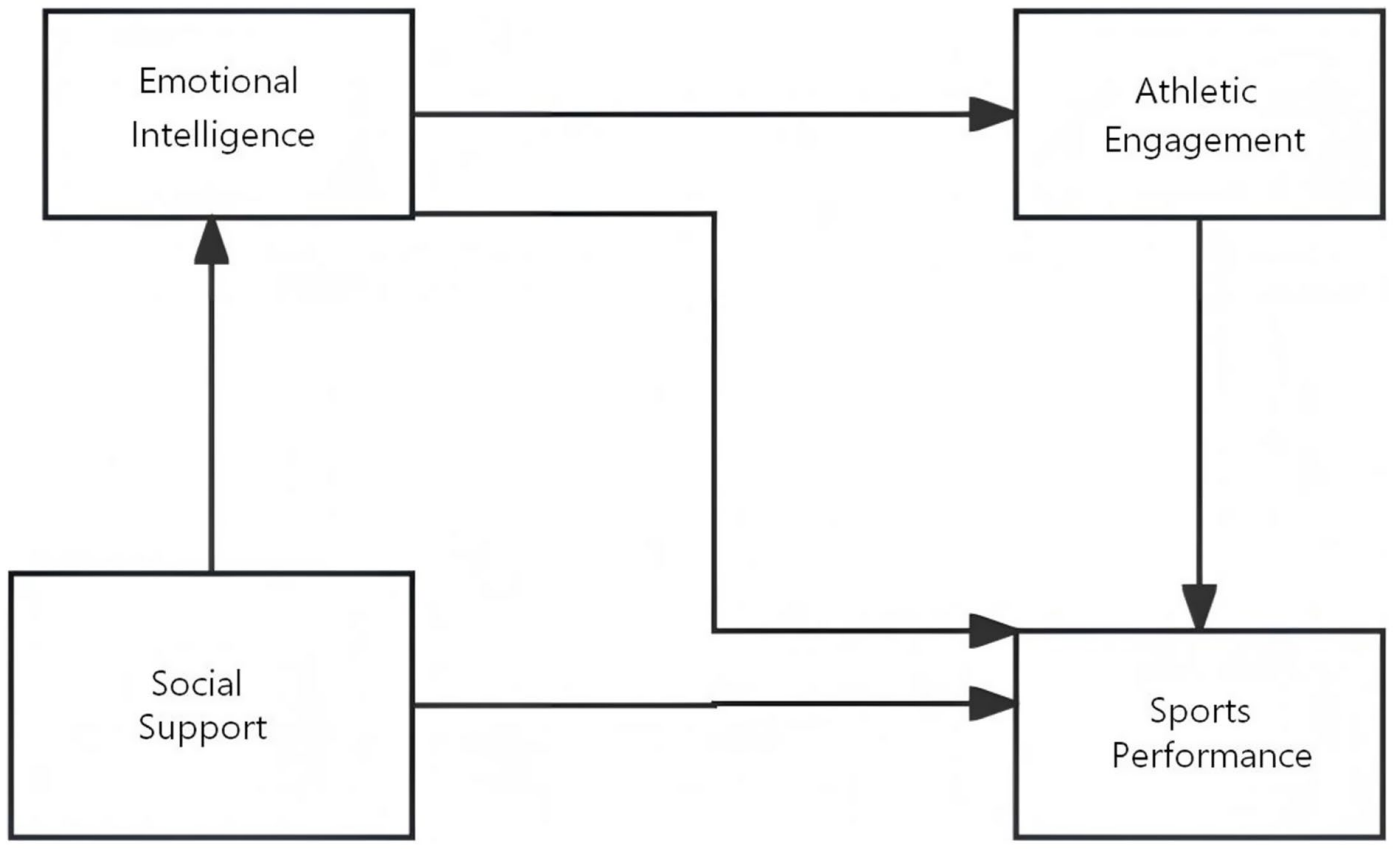
Diagram of the hypothetical model.

In the research on the relationship between social support and sports performance, emotional intelligence and athletic engagement have been proven to play significant mediating roles, respectively. Social support not only acts directly on sports performance but also indirectly promotes athletes’ sports performance by improving their emotional intelligence level (Xiong, 2024; Zhao, 2011). At the same time, social support can also improve athletes’ sports performance by enhancing their athletic engagement (Ren, 2023; Chang, 2024). Furthermore, emotional intelligence and athletic engagement form a chain mediating path of “psychology → behavior” between social support and sports performance: social support first improves athletes’ emotional intelligence, the enhancement of emotional intelligence further promotes the improvement of their athletic engagement level, and finally transforms into better sports performance through the accumulation of athletic engagement. Existing studies have initially confirmed the existence of this chain mediating mechanism in the athlete group (Ye et al., 2016; Wang C., 2024). Based on this, we propose Hypothesis H5: Emotional intelligence and athletic engagement have a chain mediating effect between social support and the sports performance of high-level college volleyball athletes, forming a path of “social support → emotional intelligence → athletic engagement → sports performance” (Figure 1).

## 3 Methodology

### 3.1 Research subjects

This study investigates the impact of social support on the athletic performance of high-level volleyball players in universities, and explores the chain-mediating effect of emotional intelligence and athletic engagement on the relationship between social support and athletic performance. The participants are athletes from 39 teams (21 men’s teams and 18 women’s teams) participating in the 2024–2025 China University Volleyball League (High-Level Group). This study employed a convenience sampling method. Participants were college volleyball athletes from China University Volleyball League. As questionnaires were completed voluntarily online, the response rate was not available. The sample covers athletes with different genders, ages and training levels, which can basically represent the target population.

### 3.2 Research methods

#### 3.2.1 Literature review method

During the research process, based on the research objectives and content, relevant literature was searched using databases such as CNKI, VIP, PubScholar using keywords such as “social support,” “emotional intelligence,” “sports engagement,” “sports performance,” “high-level college athletes,” and “chain mediation.” The relevant literature was read and analyzed to understand the research findings in various fields and to summarize the definitions of core concepts and explanations of core theories.

#### 3.2.2 Questionnaire survey method

The questionnaire survey method is a quantitative research tool that collects data through a set of scientifically designed standardized questions. This study adopted the questionnaire survey method, selecting a mature scale as the research tool. A combination of online and offline methods was used to distribute and collect questionnaires from athletes of 39 participating teams in the 2024–2025 China University Volleyball League (High-Level Group), collecting the necessary research data.

#### 3.2.3 Mathematical statistics method

This paper primarily uses Excel 2021 and SPSS 26.0 software to organize and analyze the questionnaire survey data, test the reliability and validity of the questionnaire, and analyze the basic information of the survey participants, demographic differences, and correlation analysis. Simultaneously, Amos software was used to construct a SEM structural equation model to test and analyze the relationships between variables in the model, providing data support for this study.

### 3.3 Research instruments

#### 3.3.1 A social support scale

A social support scale was used to measure the social support of high-level volleyball players in universities, based on the “Social Support Rating Scale for College Students” developed by Ye Yuemei and Dai Xiaoyang. The scale consists of three dimensions: subjective support, objective support, and support utilization, with a total of 17 items. A 5-point Likert scale (1 = completely disagree, 5 = completely agree) was used, with higher scores indicating a higher perceived level of social support. This scale has been widely validated among university students and has a good foundation of reliability and validity.

#### 3.3.2 Emotional intelligence scale

The “Volleyball Players’ Emotional Intelligence Scale” developed by Wu Qiong was used to measure the emotional intelligence level of high-level volleyball players in universities. This scale includes four dimensions: emotion perception, emotion expression, emotion evaluation, and emotion regulation, with a total of 28 items. The scale uses a 5-point Likert scoring system (1 = completely disagree, 5 = completely agree), with higher scores indicating better emotional intelligence development. This scale was specifically developed for volleyball players, and the items are closely related to the context of volleyball, exhibiting high content validity.

#### 3.3.3 Sports engagement scale

The Chinese version of the Athlete Engagement Questionnaire (AEQ), translated and revised by Ye et al. (2016), was used. This questionnaire was developed by Lonsdale et al. (2007). The scale consists of four dimensions: confidence, dedication, vitality, and enthusiasm, with a total of 16 items. A 5-point Likert scale (1 = completely disagree, 5 = completely agree) was used. Higher scores indicate a higher level of engagement in training and competition. This scale is widely used in the international field of sports psychology, and the Chinese version showed good reliability and validity.3.3.3 Sports Engagement Scale The Chinese version of the Athlete Engagement Questionnaire (AEQ), translated and revised by Ye et al. (2016), was used. This questionnaire was developed by Lonsdale et al. (2007). The scale consists of four dimensions: confidence, dedication, vitality, and enthusiasm, with a total of 16 items. A 5-point Likert scale (1 = completely disagree, 5 = completely agree) was used. Higher scores indicate a higher level of engagement in training and competition. This scale is widely used in the international field of sports psychology, and the Chinese version showed good reliability and validity.

### 3.4 Test of reliability and validity

Since the study participants were high-level volleyball players from universities, a small-scale pre-survey was conducted before the formal administration of the scales to ensure their applicability and reliability in this research context. Fifty athletes from the volleyball teams of Shenyang University of Chemical Technology, Jilin University, and Beijing Sport University were randomly selected as test subjects. Cronbach’s *α* coefficient was used to test internal consistency reliability, and confirmatory factor analysis (CFA) was used to test the construct validity of the scales. The results showed that the Cronbach’s α coefficients for the three scales were 0.799, 0.901, and 0.834, respectively, all greater than 0.7, indicating good internal consistency reliability. All fit indices in the confirmatory factor analysis were within acceptable ranges, indicating that each scale has good construct validity in this study sample and is suitable for a formal survey of high-level volleyball players from universities (Tables 1–3).

**Table 1:** Test of reliability and validity of college volleyball players social support scale.

Cronbach’s alpha	*χ*^2^/df	RMSEA	RMR	CFI	NNFI
0.799	0.883	0.000	0.020	1	1

**Table 2:** Test of reliability and validity of emotional intelligence scale for college volleyball players.

Cronbach’s alpha	*χ*^2^/df	RMSEA	RMR	CFI	NNFI
0.901	1.293	0.077	0.036	1	1

**Table 3:** Test of reliability and validity of college volleyball players’ sports input scale.

Cronbach’s alpha	*χ*^2^/df	RMSEA	RMR	CFI	NNFI
0.834	1.302	0.078	0.043	1	1

## 4 Research results

### 4.1 Common method Bias test

Common method bias refers to the artificial covariance between predictor and criterion variables caused by the same data source or raters, the same measurement environment, item context, and the characteristics or content of the questionnaire itself. This can affect the validity and validity of research results. Therefore, a common method bias test should be performed on the questionnaire data before the research begins to ensure the validity of the data and lay the foundation for subsequent research (Table 4).

**Table 4:** Results of common method deviation tests.

Factor numbering	Characteristic roots			Rotational forward difference explanation rate			Rotational variance explained		
	Characteristic roots	Variance explained%	Grand total%	Characteristic roots	Variance explained%	Characteristic roots	Characteristic roots	Variance explained%	Characteristic roots
1	15.398	25.243	25.243	15.398	25.243	25.243	3.351	5.493	5.493
2	1.861	3.051	28.294	1.861	3.051	28.294	2.720	4.460	9.953
3	1.709	2.801	31.095	1.709	2.801	31.095	2.588	4.242	14.195
4	1.647	2.701	33.796	1.647	2.701	33.796	2.484	4.073	18.268
5	1.595	2.616	36.411	1.595	2.616	36.411	2.417	3.962	22.230
6	1.545	2.533	38.944	1.545	2.533	38.944	2.331	3.822	26.052
7	1.407	2.306	41.251	1.407	2.306	41.251	2.324	3.810	29.863
8	1.385	2.271	43.522	1.385	2.271	43.522	2.082	3.413	33.276
9	1.377	2.257	45.778	1.377	2.257	45.778	2.031	3.330	36.606
10	1.318	2.161	47.940	1.318	2.161	47.940	1.954	3.203	39.809
11	1.262	2.069	50.009	1.262	2.069	50.009	1.948	3.194	43.002
12	1.211	1.985	51.994	1.211	1.985	51.994	1.925	3.155	46.157
13	1.138	1.866	53.860	1.138	1.866	53.860	1.901	3.116	49.273
14	1.089	1.785	55.645	1.089	1.785	55.645	1.802	2.954	52.227
15	1.060	1.737	57.382	1.060	1.737	57.382	1.760	2.886	55.113
16	1.040	1.705	59.088	1.040	1.705	59.088	1.730	2.836	57.950
17	1.022	1.675	60.762	1.022	1.675	60.762	1.677	2.750	60.700

In this study, the Harman one-way statistical control method was used, with the percentage of variance explained by the first factor as the criterion for judgment (Zhao Juan,2011). The results showed that the variance explained by the first factor was 25.243%, which did not exceed the critical criterion of 40%, indicating that there was no obvious common method bias and further analysis was possible.

### 4.2 A descriptive analysis of social support, emotional intelligence, and athletic engagement among high-level volleyball players in universities

#### 4.2.1 A descriptive analysis of social support for high-level volleyball players in universities

In terms of social support, the average score was 59.210, and the median was 63.000, significantly higher than the average. Combined with a standard deviation of 11.098, this indicates that high-level volleyball players in universities generally receive a certain level of social support, but significant differences exist between individuals. Interviews revealed that universities place considerable importance on volleyball players, providing them with training facilities, equipment, and other material resources, assigning professional coaches, and offering some flexibility in their academic schedules, thus laying a solid foundation for overall social support (Table 5).

**Table 5:** Descriptive analysis of social support for high-level volleyball athletes in universities (*n =* 518).

Heading	Minimum value	Maximum value	Average value	Standard deviation	Median
Subjective support	6.000	24.000	17.210	3.713	18.000
Objective support	8.000	29.000	21.006	4.414	22.000
Support utilization	7.000	29.000	20.994	4.684	22.000
Total Social Support Score	28.000	79.000	59.210	11.098	63.000

#### 4.2.2 A descriptive analysis of the emotional intelligence of high-level volleyball players in universities

In terms of social support, the average total score was 98.261, and the median was 104.000, which was significantly higher than the average. Combined with the standard deviation of 17.512, this indicates that the overall emotional intelligence performance of high-level volleyball players in universities is acceptable, but there are significant differences among individuals (Table 6).

**Table 6:** Descriptive analysis of emotional intelligence of high-level volleyball players in universities (*n =* 518).

Heading	Minimum value	Maximum value	Average value	Standard deviation	Median
Emotional perception	12.000	37.000	27.880	5.643	30.000
Emotional expression	9.000	29.000	20.917	4.322	22.000
Emotional rating	6.000	25.000	17.882	3.666	19.000
Emotion Regulation	14.000	42.000	31.581	6.184	33.000
Total score of emotional intelligence	50.000	120.000	98.261	17.512	104.000

#### 4.2.3 Descriptive analysis of the sports input of high-level volleyball players in universities

In terms of athletic engagement, the average total score was 56.050, and the median was 59.000, which was significantly higher than the average. Combined with the standard deviation of 10.315, this indicates that the overall athletic engagement of high-level volleyball players in universities is in the middle range, with some differences between individuals. Some athletes have a high level of engagement, while others still have room for improvement (Table 7).

**Table 7:** Descriptive analysis of high-level volleyball players’ sports investment in universities (*n =* 518).

Heading	Minimum value	Maximum value	Average value	Standard deviation	Median
Confidence	4.000	20.000	14.033	3.093	15.000
Dedication	4.000	20.000	14.280	3.041	15.000
Vitality	5.000	20.000	13.863	3.126	14.500
Enthusiasm	5.000	20.000	13.875	3.175	14.000
Total score of sports input	23.000	72.000	56.050	10.315	59.000

### 4.3 Difference analysis of social support, emotional intelligence and athletic engagement of high-level college volleyball athletes

#### 4.3.1 Difference analysis of social support among different athlete groups

It can be seen from the data in the Table 8 that there are no significant differences in subjective support, objective support, support utilization, and total score of social support among high-level college volleyball athletes of different sexes (*p* > 0.05). This indicates that both male and female volleyball athletes have similar levels of social support and support utilization.

**Table 8:** Analysis of the differences of social support among high-level volleyball athletes of different sexes in colleges and universities (*n =* 518).

Indicator	Male (*n =* 288)	Female (*n =* 230)	*t*	*p*
Subjective support	17.23 ± 3.76	17.19 ± 3.66	0.128	0.898
Objective support	20.90 ± 4.16	21.13 ± 4.73	−0.594	0.553
Support utilization	20.91 ± 4.85	21.10 ± 4.48	−0.440	0.660
Total score of social support	59.05 ± 11.13	59.42 ± 11.08	−0.379	0.705

It can be seen from the data in the Table 9 that there is a significant difference in subjective support among high-level college volleyball athletes of different ages at the 0.05 level (*p* = 0.017 < 0.05). LSD post-hoc test shows that the 18-19-year-old group has the highest subjective support.

**Table 9:** Analysis of the differences of social support among high-level volleyball players of different ages in colleges and universities (*n =* 518).

Indicator	18–19 age (*n =* 102)	20–21 age (*n =* 154)	22–23 age (*n =* 153)	24 years and older (*n =* 109)	*F*	*p*
Subjective Support	18.22 ± 2.98	16.76 ± 3.54	17.04 ± 3.89	17.15 ± 4.18	3.415	0.017*
Objective Support	22.26 ± 3.79	20.33 ± 4.25	20.83 ± 4.59	21.03 ± 4.74	4.119	0.007**
Support Utilization	21.89 ± 3.90	21.28 ± 5.04	20.08 ± 4.51	21.04 ± 4.92	3.440	0.017*
Total score of social support	62.37 ± 8.78	58.37 ± 11.22	57.95 ± 11.16	59.21 ± 12.30	3.774	0.011*

*p < 0.05, **p < 0.01.

It can be seen from the data in the Table 10 that there are no significant differences in subjective support, objective support, support utilization, and total score of social support among high-level college volleyball athletes of different sports grades (*p* > 0.05). This indicates that Master Athletes, First-class Athletes, and Second-class Athletes have similar levels of social support and support utilization.

**Table 10:** Analysis of the differences of social support among high-level volleyball players of different sports grades in colleges and universities (*n =* 518).

Indicator	Master athlete (*n =* 145)	First-class athlete (*n =* 262)	Second-class athlete (*n =* 111)	*F*	*p*
Subjective Support	17.12 ± 3.89	17.22 ± 3.70	17.32 ± 3.53	0.090	0.914
Objective Support	21.55 ± 4.16	20.84 ± 4.69	20.69 ± 4.01	1.584	0.206
Support Utilization	21.61 ± 4.80	20.53 ± 4.63	21.27 ± 4.58	2.742	0.065
Total score of social support	60.28 ± 11.29	58.59 ± 11.26	59.28 ± 10.42	1.092	0.336

*p < 0.05, **p < 0.01.

#### 4.3.2 Difference analysis of emotional intelligence among different athlete groups

It can be seen from the data in the Table 11 that there are no significant differences in emotional perception, emotional expression, emotional evaluation, emotional regulation, and total score of emotional intelligence among high-level college volleyball athletes of different sexes (*p* > 0.05). This indicates that both male and female volleyball athletes have similar levels of emotional intelligence.

**Table 11:** Analysis of the differences of emotional intelligence of high-level volleyball players in different sexes (*n =* 518).

Indicator	Male (*n =* 288)	Female (*n =* 230)	*t*	*p*
Emotional Perception	27.91 ± 5.53	27.84 ± 5.79	0.133	0.895
Emotional Expression	20.92 ± 4.34	20.91 ± 4.30	0.039	0.969
Emotional Evaluation	17.93 ± 3.71	17.82 ± 3.62	0.335	0.738
Emotional Regulation	31.69 ± 6.36	31.44 ± 5.96	0.467	0.641
Total score of emotional intelligence	98.46 ± 17.66	98.01 ± 17.35	0.287	0.774

It can be seen from the data in the Table 12 that there are no significant differences in emotional perception and emotional regulation among high-level college volleyball athletes of different ages (*p* > 0.05), while there are significant differences in emotional expression, emotional evaluation, and total score of emotional intelligence among high-level college volleyball athletes of different ages (*p* < 0.05).

**Table 12:** Analysis of the differences of emotional intelligence of high-level volleyball players in different ages (*n =* 518).

Indicator	18–19 years old (*n =* 102)	20–21 years old (*n =* 154)	22–23 years old (*n =* 153)	24 years old and above (*n =* 109)	*F*	*p*
Emotional Perception	28.92 ± 4.69	27.67 ± 6.53	27.10 ± 5.39	28.30 ± 5.33	2.434	0.064
Emotional Expression	22.42 ± 3.59	20.33 ± 4.88	20.93 ± 4.01	20.32 ± 4.24	5.919	0.001**
Emotional Evaluation	18.94 ± 3.62	17.09 ± 3.58	17.89 ± 3.49	18.00 ± 3.85	5.400	0.001**
Emotional Regulation	32.55 ± 4.71	30.86 ± 6.38	31.22 ± 6.45	32.20 ± 6.63	2.088	0.101
Total Score of emotional intelligence	102.83 ± 14.03	95.95 ± 19.35	97.14 ± 17.11	98.83 ± 17.66	3.512	0.015*

*p < 0.05, **p < 0.01.

It can be seen from the data in the Table 13 that there are no significant differences in emotional perception, emotional expression, emotional evaluation, emotional regulation, and total score of emotional intelligence among high-level college volleyball athletes of different sports grades (*p* > 0.05). This indicates that Master Athletes, First-class Athletes, and Second-class Athletes have similar levels of emotional intelligence.

**Table 13:** Analysis of the differences of emotional intelligence among high-level volleyball players of different sports grades (*n =* 518).

Indicator	Master athlete (*n =* 145)	First-class athlete (*n =* 262)	Second-class athlete (*n =* 111)	*F*	*p*
Emotional Perception	27.98 ± 5.65	27.57 ± 5.84	28.49 ± 5.12	1.062	0.346
Emotional Expression	21.26 ± 4.00	20.83 ± 4.60	20.68 ± 4.05	0.667	0.514
Emotional Evaluation	18.28 ± 3.62	17.89 ± 3.60	17.34 ± 3.85	2.078	0.126
Emotional Regulation	32.05 ± 5.83	31.58 ± 6.69	30.96 ± 5.33	0.966	0.381
Total Score of Emotional Intelligence	99.57 ± 16.98	97.87 ± 18.45	97.47 ± 15.89	0.579	0.561

#### 4.3.3 Difference analysis of athletic engagement among different athlete groups

It can be seen from the data in the Table 14 that there are no significant differences in confidence, dedication, vitality, enthusiasm, and total score of athletic engagement among high-level college volleyball athletes of different sexes (p > 0.05). This indicates that both male and female volleyball athletes have similar levels of athletic engagement.

**Table 14:** Analysis of the differences of high-level volleyball athletes’ athletic engagement in different sexes (*n =* 518).

Indicator	Male (*n =* 288)	Female (*n =* 230)	*t*	*p*
Confidence	14.15 ± 3.14	13.89 ± 3.03	0.930	0.353
Dedication	14.25 ± 3.22	14.32 ± 2.81	−0.279	0.780
Vitality	13.87 ± 3.13	13.85 ± 3.13	0.070	0.944
Enthusiasm	13.81 ± 3.11	13.96 ± 3.26	−0.553	0.581
Total score of athletic engagement	56.07 ± 10.66	56.03 ± 9.88	0.047	0.962

It can be seen from the data in the Table 15 that there are no significant differences in dedication, vitality, enthusiasm, and total score of athletic engagement among high-level college volleyball athletes of different ages (*p* > 0.05), while there is a significant difference in confidence among high-level college volleyball athletes of different ages at the 0.05 level (*p* = 0.014 < 0.05). LSD post-hoc test shows that athletes aged 18–19 have the strongest confidence.

**Table 15:** Analysis of the differences of high-level volleyball athletes’ athletic engagement in different ages (*n =* 518).

Indicator	18–19 years old (*n =* 102)	20–21 years old (*n =* 154)	22–23 years old (*n =* 153)	24 years old and above (*n =* 109)	*F*	*p*
Confidence	14.99 ± 2.28	13.73 ± 3.16	13.75 ± 3.04	13.97 ± 3.56	4.294	0.005**
Dedication	14.71 ± 2.69	14.00 ± 3.34	14.16 ± 3.19	14.45 ± 2.64	1.301	0.273
Vitality	14.05 ± 3.00	13.80 ± 3.27	13.73 ± 3.21	13.97 ± 2.94	0.284	0.837
Enthusiasm	14.13 ± 2.40	13.67 ± 3.28	13.73 ± 3.12	14.14 ± 3.70	0.792	0.499
Total score of athletic engagement	57.87 ± 8.15	55.19 ± 10.88	55.35 ± 10.92	56.53 ± 10.33	1.734	0.159

*p < 0.05, **p < 0.01.

It can be seen from the data in the Table 16 that there are no significant differences in confidence, dedication, vitality, enthusiasm, and total score of athletic engagement among high-level college volleyball athletes of different sports grades (p > 0.05). This indicates that Master Athletes, First-class Athletes, and Second-class Athletes have similar levels of athletic engagement.

**Table 16:** Analysis of the differences of athletic engagement of high-level volleyball players in different sports grades (*n =* 518).

Indicator	Master athlete (*n =* 145)	First-class athlete (*n =* 262)	Second-class athlete (*n =* 111)	*F*	*p*
Confidence	14.03 ± 2.91	13.98 ± 3.11	14.14 ± 3.31	0.103	0.902
Dedication	14.66 ± 2.67	14.09 ± 3.33	14.23 ± 2.75	1.623	0.198
Vitality	14.30 ± 3.19	13.71 ± 3.07	13.64 ± 3.13	2.029	0.133
Enthusiasm	14.19 ± 3.11	13.67 ± 3.30	13.94 ± 2.93	1.287	0.277
Total Score of Athletic Engagement	57.19 ± 9.74	55.46 ± 10.72	55.95 ± 10.03	1.312	0.270

*p < 0.05, **p < 0.01.

### 4.4 Correlation analysis of social support, emotional intelligence, athletic engagement and sports performance of high-level college volleyball athletes

#### 4.4.1 Correlation analysis of social support, emotional intelligence, athletic engagement with sports performance respectively

It can be seen from the data in the Table 17 that the Pearson correlation coefficients of subjective support, objective support, support utilization, and total score of social support with sports performance are 0.635, 0.697, 0.658, and 0.767 respectively, all reaching extremely significant levels (*p* < 0.01). This indicates that the higher the level of each dimension of social support and the overall social support, the better the sports performance of high-level college volleyball athletes.

**Table 17:** Pearson test of association between social support, emotional intelligence and athletic engagement with sports performance.

Indicator	Sports performance
Subjective Support	0.635**
Objective Support	0.697**
Support Utilization	0.658**
Social Support	0.767**
Emotional Perception	0.716**
Emotional Expression	0.681**
Emotional Evaluation	0.617**
Emotional Regulation	0.721**
Emotional Intelligence	0.783**
Confidence	0.609**
Dedication	0.631**
Vitality	0.671**
Enthusiasm	0.626**
Athletic Engagement	0.765**

*p < 0.05, **p < 0.01.

Social support, emotional intelligence, and athletic engagement, as well as their subdimensions, are positively correlated with sports performance, with emotional intelligence showing the strongest correlation, followed by social support and then athletic engagement.

#### 4.4.2 Correlation analysis between social support, emotional intelligence and athletic engagement

It can be seen from the data in the Table 18 that among the dimensions of social support, subjective support, objective support, and support utilization are all extremely significantly positively correlated with each other (p < 0.01). Moreover, their correlation coefficients with the total score of social support are 0.841, 0.879, and 0.875, respectively. This indicates that the various dimensions of social support are closely connected and jointly form the overall structure of social support, and changes in any single dimension will have a significant impact on the total score of social support.

**Table 18:** Pearson test of social support, emotional intelligence and athletic engagement.

Indicator	1	2	3	4	5	6	7	8	9	10	11	12	13	14
Subjective Support (1)	1													
Objective Support(2)	0.636**	1												
Support Utilization(3)	0.599**	0.635**	1											
Social Support (4)	0.841**	0.879**	0.875**	1										
Emotional Perception (5)	0.642**	0.689**	0.680**	0.776**	1									
Emotional Expression(6)	0.624**	0.675**	0.678**	0.763**	0.727**	1								
Emotional Evaluation (7)	0.617**	0.646**	0.643**	0.735**	0.677**	0.640**	1							
Emotional Regulation(8)	0.659**	0.740**	0.659**	0.793**	0.750**	0.717**	0.662**	1						
Emotional Intelligence(9)	0.723**	0.785**	0.754**	0.872**	0.908**	0.868**	0.820**	0.910**	1					
Confidence(10)	0.664**	0.632**	0.567**	0.713**	0.645**	0.619**	0.593**	0.642**	0.712**	1				
Dedication(11)	0.545**	0.590**	0.615**	0.676**	0.686**	0.627**	0.625**	0.677**	0.746**	0.584**	1			
Vitality(12)	0.571**	0.617**	0.596**	0.688**	0.641**	0.625**	0.590**	0.640**	0.710**	0.560**	0.585**	1		
Enthusiasm(13)	0.632**	0.634**	0.585**	0.711**	0.659**	0.623**	0.611**	0.613**	0.710**	0.557**	0.660**	0.560**	1	
Athletic Engagement(14)	0.727**	0.746**	0.712**	0.841**	0.793**	0.752**	0.729**	0.775**	0.867**	0.813**	0.850**	0.816**	0.839**	1

### 4.5 Analysis of the mediating relationship among social support, emotional intelligence, athletic engagement and sports performance of high-level college volleyball athletes

Analysis of the chain mediating relationship of emotional intelligence and athletic engagement between social support and sports performance.

It can be seen from the data in the Tables 19, 20 that the 95% confidence intervals of the effect values of the direct effect (Social Support → Sports Performance) and all paths in the indirect effect process (Social Support → Emotional Intelligence, Social Support → Athletic Engagement, Emotional Intelligence → Athletic Engagement, Emotional Intelligence → Sports Performance, Athletic Engagement → Sports Performance) do not contain 0, and all *p*-values are less than 0.001, indicating that these effects are all highly statistically significant.

**Table 19:** Fit index of chain mediation model between emotional intelligence and athletic engagement (overall).

Fit index	*χ*^2^/df	RMSEA	IFI	CFI	NFI	GFI
Recommended range	<3	<0.08	>0.9	>0.9	>0.9	>0.9
Result	1.968	0.076	0.972	0.972	0.933	0.969

**Table 20:** Chain mediation analysis process summary of social support, emotional intelligence, athletic engagement and sports performance (overall).

Effect	Item	Effect value	SE	*z*-value/*t*-value	*p*-value	95% CI	
						Lower limit	Upper limit
Direct Effect	Social Support → Sports Performance	0.263	0.056	4.669	0.000	0.152	0.373
Indirect Effect Process	Social Support → Emotional Intelligence	0.872	0.022	40.517	0.000	0.830	0.915
	Social Support → Athletic Engagement	0.352	0.042	8.355	0.000	0.269	0.435
	Emotional Intelligence → Athletic Engagement	0.560	0.042	13.308	0.000	0.478	0.643
	Emotional Intelligence → Sports Performance	0.330	0.061	5.383	0.000	0.209	0.450
	Athletic Engagement → Sports Performance	0.258	0.055	4.677	0.000	0.150	0.367
Total Effect	Social Support → Sports Performance	0.767	0.028	27.186	0.000	0.712	0.823

At the same time, the Bootstrap sampling test method was used to study the mediating effect with 5,000 sampling times, and the results are shown in Table 21.

**Table 21:** Chain mediation tests of social support, emotional intelligence, athletic engagement and sports performance (overall).

Item	Effect value	SE	*z*-value/*t*-value	*p*-value	95% CI	
					Lower limit	Upper limit
Social Support → Emotional Intelligence → Sports Performance	0.287	0.051	5.666	0.000	0.190	0.389
Social Support → Athletic Engagement → Sports Performance	0.091	0.022	4.147	0.000	0.050	0.137
Social Support → Emotional Intelligence → Athletic Engagement → Sports Performance	0.126	0.027	4.727	0.000	0.073	0.179

The establishment of the chain mediation indicates that emotional intelligence and athletic engagement play a chain mediating role between social support and sports performance of high-level college volleyball athletes (Table 22).

**Table 22:** Chain mediation analysis process summary of social support, emotional intelligence, athletic engagement and sports performance (men).

Effect	Item	Effect value	SE	*z*-value/*t*-value	*p*-value	95% CI	
						Lower limit	Upper limit
Direct Effect	Social Support → Sports Performance	0.307	0.073	4.206	0.000	0.163	0.450
Indirect Effect Process	Social Support → Emotional Intelligence	0.864	0.030	28.340	0.000	0.804	0.923
	Social Support → Athletic Engagement	0.305	0.055	5.546	0.000	0.197	0.413
	Emotional Intelligence → Athletic Engagement	0.635	0.055	11.603	0.000	0.527	0.743
	Emotional Intelligence → Sports Performance	0.378	0.084	4.516	0.000	0.213	0.542
	Athletic Engagement → Sports Performance	0.218	0.075	2.923	0.004	0.071	0.365
Total Effect	Social Support → Sports Performance	0.819	0.039	20.887	0.000	0.742	0.896

It can be seen from the data in the table that among the group of male high-level college volleyball athletes, the 95% confidence intervals of the effect values of the direct effect and all paths in the indirect effect process do not contain 0, and all p-values are less than 0.05, indicating that these effects are all highly statistically significant.

The Bootstrap sampling test method was used to study the mediating effect with 5,000 sampling times, and the results are shown in Table 23.

**Table 23:** Chain mediation tests of social support, emotional intelligence, athletic engagement and sports performance (men).

Item	Effect value	SE	*z*-value/*t*-value	*p*-value	95% CI	
					Lower limit	Upper limit
Social Support → Emotional Intelligence → Sports Performance	0.326	0.066	4.975	0.000	0.184	0.441
Social Support → Athletic Engagement → Sports Performance	0.067	0.024	2.802	0.005	0.020	0.114
Social Support → Emotional Intelligence → Athletic	0.120	0.038	3.110	0.002	0.039	0.189

The establishment of the chain mediation indicates that emotional intelligence and athletic engagement play a chain mediating role between social support and sports performance of male high-level college volleyball athletes.

It can be seen from the data in the Table 24 that both the direct effect and the total effect indicate a significant positive correlation between social support and sports performance, and the total effect (0.707) is much larger than the direct effect (0.251), indicating that the impact of social support on sports performance is partially achieved indirectly through other variables.

**Table 24:** Chain mediation analysis process of social support, emotional intelligence, athletic engagement and sports performance (women).

Effect	Item	Effect value	SE	*z*-value/*t*-value	*p*-value	95% CI	
						Lower limit	Upper limit
Direct Effect	Social Support → Sports Performance	0.251	0.084	2.996	0.003	0.086	0.416
Indirect Effect Process	Social Support → Emotional Intelligence	0.884	0.030	29.568	0.000	0.825	0.943
	Social Support → Athletic Engagement	0.436	0.066	6.647	0.000	0.307	0.566
	Emotional Intelligence → Athletic Engagement	0.439	0.066	6.648	0.000	0.309	0.570
	Emotional Intelligence → Sports Performance	0.245	0.084	2.898	0.004	0.078	0.411
	Athletic Engagement → Sports Performance	0.291	0.078	3.749	0.000	0.138	0.444
Total Effect	Social Support → Sports Performance	0.707	0.037	18.870	0.000	0.634	0.781

The Bootstrap sampling test method was used to study the mediating effect with 5,000 sampling times. The results show that emotional intelligence and athletic engagement play a chain mediating role between social support and sports performance of female high-level college volleyball athletes (Table 25).

**Table 25:** Chain mediation tests of social support, emotional intelligence, athletic engagement and sports performance (women).

Item	Effect value	SE	*z*-value/*t*-value	*p*-value	95% CI	
					Lower limit	Upper limit
Social Support → Emotional Intelligence → Sports Performance	0.216	0.073	2.975	0.003	0.101	0.388
Social Support → Athletic Engagement → Sports Performance	0.127	0.043	2.920	0.003	0.061	0.230
Social Support → Emotional Intelligence → Athletic Engagement → Sports Performance	0.113	0.037	3.079	0.002	0.056	0.200

## 5 Discussion

### 5.1 Discussion on the current situation of social support, emotional intelligence and athletic engagement of high-level college volleyball athletes

In this study, through the investigation of social support, emotional intelligence, and athletic engagement of high-level college volleyball athletes, it was found that high-level college volleyball athletes obtained certain social support in their daily training and competitions, showed good emotional intelligence, possessed the ability to perceive, express, evaluate, and regulate emotions, and their athletic engagement was at a moderate level, with a balanced development of confidence, dedication, vitality, and enthusiasm for sports. At the same time, there were no significant differences in social support, emotional intelligence, and athletic engagement among high-level college volleyball athletes of different genders and sports levels, but there were differences of varying degrees among different ages (Ren, 2023; Lu, 2024). This research result is consistent with the research results of Ren Guangxi, Lu Pukun, and other scholars.

### 5.2 Discussion on the correlation among social support, emotional intelligence, athletic engagement and sports performance of high-level college volleyball athletes

In the research process, Pearson correlation and linear regression were used to analyze the correlation between social support, emotional intelligence, athletic engagement, and sports performance of high-level college volleyball athletes. The results showed that social support, emotional intelligence, athletic engagement, and their sub-dimensions were positively correlated with the sports performance of high-level college volleyball athletes, respectively. When social support, emotional intelligence, and athletic engagement increased respectively, sports performance also improved, and emotional intelligence had the strongest correlation with sports performance. In addition, social support, emotional intelligence, athletic engagement, and their sub-dimensions were also pairwise correlated (Zhai et al., 2019; Zhang, 2022). This research result is consistent with the research results of Zhai Lei, Zhang Jiaqi, and other scholars.

The significant positive association between social support and competitive sports performance can be deeply interpreted from mature psychological theoretical perspectives rather than superficial descriptive explanations. Based on the Conservation of Resources Theory (COR), social support serves as stable and valuable external psychological resources for athletes. In high-intensity training and fierce competitive situations, athletes often face stress, anxiety, fatigue and resource consumption. Adequate social support from coaches, teammates and families can effectively buffer competitive pressure, reduce emotional exhaustion and avoid the loss of internal psychological resources, so as to maintain a stable and positive psychological state (Wang, 2024). Meanwhile, from the perspective of Self-Determination Theory (SDT), high-quality social support can effectively satisfy athletes’ three basic psychological needs: autonomy, competence and relatedness. The satisfaction of basic needs can stimulate internal motivation, optimize emotional experience, and lay a solid psychological foundation for the improvement of emotional intelligence.

Furthermore, the chain mediating mechanism of social support → emotional intelligence → athletic engagement → sports performance reveals a complete internal transmission path of psychological effects. Social support does not directly improve sports performance, but indirectly acts on performance through successive psychological processes (Wen et al., 2004). First, positive social support shapes a safe and supportive training atmosphere, which helps athletes correctly perceive, understand and manage their own emotions, thus significantly improving their emotional intelligence level. Second, athletes with higher emotional intelligence are better at regulating negative emotions, relieving competitive pressure, maintaining attention and enthusiasm in training, and then showing higher vitality, dedication and absorption in athletic engagement. Finally, sustained high-level athletic engagement can promote efficient training input, a stable competitive state and better on-the-spot performance in competitions through stable performance.

### 5.3 Discussion on the mediating relationship among social support, emotional intelligence, athletic engagement and sports performance of high-level college volleyball athletes

In the research process, by constructing a structural equation and conducting model fitting, combined with the verification results of the Bootstrap sampling test method, it was found that among high-level college volleyball athletes, emotional intelligence and athletic engagement can, respectively, play a significant mediating role between social support and sports performance. At the same time, whether for male or female athletes, emotional intelligence and athletic engagement can, respectively, have a significant mediating relationship between social support and sports performance (Chang, 2024; Xiong, 2024). This research result is consistent with the research results of Chang Shi, Xiong Yijing, and other scholars. At the same time, there is a chain mediating effect of social support on sports performance, in which emotional intelligence and athletic engagement play a chain mediating role, that is, social support affects emotional intelligence, which in turn affects athletic engagement, and finally has an impact on sports performance. The chain mediating effect of female high-level college volleyball athletes is slightly higher than that of male high-level college volleyball athletes, and the stability of the male effect is slightly weaker than that of the female effect.

For high-level college volleyball athletes, material support such as professional training equipment and good training venues can provide basic conditions for them to carry out efficient training. Spiritual support such as coaches’ encouragement, teammates’ company and trust, and family’s understanding and support can give athletes emotional comfort and motivation. These supports create a good training and competition environment for athletes, enabling them to devote themselves to volleyball wholeheartedly. Sufficient social support makes athletes feel valued and cared for, thereby stimulating positive emotional experiences (Zhang, 2022). When athletes’ basic psychological needs are met, their intrinsic motivation is stimulated, making them more actively engaged in volleyball. Intrinsic motivation is an important driving force for athletic engagement. Athletes with intrinsic motivation will consciously overcome difficulties and continuously improve their sports skills and levels (Guo and Yang, 2021). The enhancement of this intrinsic motivation further promotes athletic engagement, which in turn has a positive impact on sports performance. The gender difference in the chain mediating effect is due to the fact that in the socialization process of the growth of high-level college athletes, social culture and education have higher expectations for women’s gender role of “relational self,” making women pay more attention to the intimacy of interpersonal relationships (Meng, 2023). In addition, women are more delicate and sensitive to emotions and have a stronger ability to perceive social support. They are more likely to obtain emotional satisfaction from the support of coaches, teammates, and family, thereby more effectively transforming this support into the improvement of emotional intelligence and the increase of athletic engagement. Men are expected to have the gender role of “independent self,” emphasizing independence and self-achievement, and have a relatively low dependence on social support. They are more inclined to solve problems through their own efforts and have relatively weak ability to perceive and utilize social support. Therefore, the impact of social support on the emotional intelligence and athletic engagement of male athletes may not be as obvious as that of female athletes, leading to slightly weaker stability of the chain mediating effect.

### 5.4 Limitations and future research directions

Several limitations of this study should be noted. Although Harman’s single-factor test indicated no serious common method variance, the relatively high correlations (0.767–0.872) among core variables should be acknowledged. These high correlations mainly originate from the close inherent theoretical relationships and inevitable conceptual overlap between social support, emotional intelligence, athletic engagement and sports performance in sport psychology. In addition, all variables were measured by single-source self-reported questionnaires, which may lead to slight potential common method variance risk. This issue does not affect the validity and reliability of the main mediating conclusions of this study. Future research can use multi-source evaluation (e.g., coach-rated sports performance, peer-rated social support) and longitudinal designs to further optimize the research effect and more accurately distinguish the independent effects of each variable.

## 6 Conclusion

Taking high-level college volleyball athletes as the research objects, this study examined the relationships among social support, emotional intelligence, athletic engagement, and sports performance. The results showed that: the overall level of the three was good, but there were significant age differences (athletes aged 18–19 performed the best in the support, emotional intelligence, and confidence dimensions), and there were no significant differences in gender and sports level; all three were significantly positively correlated with sports performance (emotional intelligence had the strongest correlation), and all were significantly positively correlated pairwise; emotional intelligence and athletic engagement, respectively, played a mediating role between social support and sports performance, and formed a chain mediating path of “social support → emotional intelligence → athletic engagement → sports performance”, which was stronger and more stable in female athletes. Suggestions: Construct a diversified support system, with schools increasing resource investment, coaches strengthening guidance, parents providing emotional support, and paying attention to age differences; incorporate emotional intelligence training into training plans and stimulate athletic engagement through goal setting; target gender differences, strengthen emotional care and stress counseling for females, and focus on emotional expression and psychological adjustment for males to improve emotional stability and concentration.

## Data Availability

The original contributions presented in the study are included in the article/supplementary material, further inquiries can be directed to the corresponding author.

## References

[ref1] Bianco T. Eklund R. C. (2001). Conceptual considerations for social support research in sport and exercise settings: the case of sport injury. J. Sport Exerc. Psychol. 23, 85–107. doi: 10.1123/jsep.23.2.85

[ref2] Chang S. (2024). A Study on the Impact of Coach-Athlete Relationship on Sports Performance Satisfaction of College Athletes in Henan Province. Henan: Henan University.

[ref4] Guo Z. Yang J. (2021). The relationship between adolescent athletes' belief in a just world and sports performance: the chain mediating role of perceived coach support and sports environment justice. J. Shenyang Sport Univ. 40, 85–93. doi: 10.12163/j.ssu.20201700

[ref6] House J. S. Umberson D. Landis K. R. (1988). Structures and processes of social support. Annu. Rev. Sociol. 1, 293–318.

[ref7] Hu X. (2017). A Study on the Impact of Social Support on Sports Performance of High-Level Taekwondo Athletes. Chengdu: Chengdu University.

[ref8] Hu W. (2025). The Relationship between Perceived Social Support and Peer Relationship of Junior High School Students: The Mediating Role and Intervention Study of Emotional Intelligence. Chengdu: Chengdu University.

[ref9] Lin L. (2024). The Impact of Emotional Intelligence on Tennis Performance of College Tennis Athletes. Chongqing: Southwest University.

[ref11] Liu Z. (2023). An Investigation on the Training Status of Male Volleyball Specialists in High Schools in Inner Mongolia Autonomous Region. Hohhot: Inner Mongolia Normal University.

[ref12] Long X. (2024). The Impact of Coaches' Leadership Behavior on College Athletes' Satisfaction with Training and Competition. Guiyang: Guizhou Minzu University.

[ref13] Lonsdale C. Hodge K. Raedeke T. D. (2007). Athlete engagement: i. a qualitative investigation of relevance and dimensions. Int. J. Sport Psychol. 38, 451–470.

[ref14] Lu P. (2024). The Impact of Perceived Social Support on Sports Engagement: The Chain Mediating Effect of Basic Psychological Needs and Self-Determination Motivation. Chongqing: Southwest University.

[ref15] Mayer J. D. Salovey P. (1997). What is Emotional Intelligence. New Jersey: Rutgers University.

[ref16] Meng Y. (2023). The Impact of Coaches' Autonomous Support on the Engagement of College Basketball Athletes. Guilin: Guangxi Normal University.

[ref17] Qiu T. (2015). The impact of perceived social support on basketball athletes' performance under stress: the mediating role of coping efficacy. J. Wuhan Sport Univ. 49, 96–100. doi: 10.15930/j.cnki.wtxb.2015.04.018

[ref18] Ren G. (2023). A Study on the Relationship between Social Support and Sports Engagement of College Football Athletes. Shanghai: Shanghai Normal University.

[ref20] Wang Z. (2024). The Relationship between Competitive Motivation and Sports Engagement of College Athletes: The Mediating Role of Sports Self-Efficacy. Huaibei: Huaibei Normal University.

[ref21] Wang C. (2024). A Study on Self-Reflection, Emotional Intelligence and Sports Engagement of Basketball Athletes in the Middle School Group of Sichuan "Gongga Cup". Chengdu: Sichuan Normal University.

[ref1001] Wen Z. L. Zhang L. Hou J. T. Liu H. Y. (2004). Testing procedure for mediating effects and its application. Acta Psychologica Sinica 36, 614–620.

[ref22] Xiong Y. (2024). A Study on the Relationship between Perceived Teacher Support, Trait Emotional Intelligence and English Performance of High School Students. Nanchang: Jiangxi Normal University.

[ref23] Yang R. (2024). The Relationship between Emotional Intelligence and Competitive State Anxiety of College Basketball Athletes. Hunan: Jishou University.

[ref24] Ye L. Wang B. Liu Z. Wu Y. Dong L. (2016). The impact of coach-athlete relationship on sports performance satisfaction: the sequential mediating role of hope and sports engagement. China Sport Sci. 36, 40–48. doi: 10.16469/j.css.201607005

[ref25] Zhai L. Li T. Sun W. (2019). The impact of grit and athlete engagement on sports performance of Sanda athletes. Hubei Sports Sci. 38, 1002–1005. doi: 10.3969/j.issn.1003-983X.2019.11.014

[ref26] Zhang Z. (2003). The relationship between human physical, emotional and intellectual cycles and sports performance. J. Shanghai Univ. Sport 6, 14–16. doi: 10.16099/j.cnki.jsus.2003.06.006

[ref27] Zhang J. (2022). A Study on the Relationship between State Anxiety, Social Support and Sports Performance of Adolescent Tennis Players in Chongqing. Chongqing: Southwest University.

[ref28] Zhang W. (2024). The Impact of Gratitude on Sports Engagement of College Volleyball Athletes. Shenyang: Shenyang Sport University.

[ref29] Zhao J. (2011). The Relationship between College Students' Internal-External Locus of Control, Perceived Social Support and School Adaptation. Guangzhou: Guangzhou University.

[ref30] Zhou R. (2009). Sports Management Research. Beijing: China Theatre Press.

